# Hospital and Institutional News

**Published:** 1918-10-19

**Authors:** 


### HOSPITAL AND INSTITUTIONAL NEWS.

### LOCAL AUTHORITIES AND A HEALTH MINISTRY.

THE Metropolitan Boroughs Standing Joint Committee has considered the effect of the proposed Ministry of Health on the London districts. The committee thinks that the Local Government Board should be the foundation of the Ministry of Health, and that the local administration of all the powers and duties necessary for the public health should be entrusted in London to the Corporation of the City of London and the Metropolitan Borough Councils. Dealing with the cost of administration, the committee considers that to prevent an undue increase in the burden falling on the ratepayers because of the increased cost of new measures or of extended schemes, adequate Exchequer grants should meet at least half the total cost. In all new and approved measures initiated by the Government affecting child life, food, or health it has either borne the whole cost or one half.

### THE GARRETT ANDERSON MEMORIAL.

THOSE responsible for the conduct of the appeal on behalf of the Elizabeth Garrett Anderson Hospital for Women, Euston Road, are as fertile in suggestion as good propagandists need to be. After successfully converting the Jubilee Fund of 1916 (for the endowment of fifty beds) into a memorial fund a year later, when Dr. Anderson died, they are now supplementing the efforts of women's colleges, the girls' schools, and the professions practised by women in general by a Tribute Sale, to be held on November 29 and 30 at the Central Hall, Westminster. The treasurer of the memorial fund is Lady Hall, who will gladly receive gifts for the sale at 144 Euston Road. The Queen has sent a number of gifts. Twenty-one or more stalls have been arranged, and Her Majesty's gifts will provide an ornament to several. Old works, white elephants, old prints, toys, produce (delightfully suggestive term, with the racy flavour of an allotment about it), and soldiers' comforts will be obtainable at separate stalls. Some prospective buyers are understood to have named prices for certain articles of which they are in search. One is a necklace of *lapis lazuli*, the stone prized, readers of Browning will remember, by the Bishop of St. Praxed's, and described in two magnificent metaphors.

### THE ALLOTMENT-HOLDERS' HARVEST.

WE must remember in these days that among the workers for that harvest for which a thanksgiving is traditionally being held, the allotment-holder has come to hold an honoured and important place. His harvest is now "home," and King Edward VII.'s Hospital, Cardiff, has begun to participate in excellent schemes whereby the allotment-holders of the district are making a gift of potatoes to their mother institution. The collections of potatoes for the hospital have been in progress, and Bargoed district and Gwaelodygarth village were among the first to return their promised quota. Since the allotment-holders in Cardiff number 6,000, a gift of 7 lb. of potatoes per head will provide the institution with this staple for six months on end. We live in a time when old proverbs are becoming better understood, and there is no truer than that which says "take care of the potatoes and the pounds will take care of themselves." At all events every pound of potatoes does something to raise the new income necessary for the enlarged hospital, and to make the scandal of the long waiting-list one of the shames of the past. Colonel Bruce-Vaughan knows this, but he knows too, as a recent admirable letter shows, the joy of hospital service, and that the pleasure even of an allotment can be increased by taking a personal pride not only in the quality of one's tubers, but in their destination and the use to which they will be put, for others.

### THE ENTERTAINMENTS TAX.

IF there is any institution concerned in the organisation of entertainments for the benefit of its funds which has not made a practice of using stamped tickets, the experience, not long ago, of the Keighley gala provides a warning. On that occasion, apparently, stamped tickets were not used, and in consequence the Board of Inland Revenue insisted on a deposit of £80. While this sum was, so to speak, in bond the interest upon it was lost by the depositors, and when it was possible to calculate the tax after the gala the sum came to £69. Application for the return of the balance was thereupon made, but not till a considerable period had elapsed was the money actually refunded. The moral, now generally recognised, we hope, is, use stamped tickets. At the same time, the system of certified returns occasions trouble, and it is to be hoped that it may be simplified, at all events in the case of performances for charity.

### SIR FRANCIS LLOYD'S FAREWELL.

LIEUT.-GENERAL SIR FRANCIS LLOYD has sent a valedictory letter to Col. Toogood, the Officer Commanding the Lewisham Military Hospital. The letter stated: "I wish to let you know how exceedingly pleased I was with everything I found in your hospital. The organisation seems to me to be as good as it is possible to find, and points to an entirely satisfactory working between the hospital staff and the Board of Guardians, who are much to be congratulated. All the kitchens were clean and the food excellent. The German prisoners in one of your wards were absolutely comfortable and satisfied; which speaks a great deal for the devotion of your medical officers, nurses, and orderlies. I feel perfectly satisfied in leaving the care of this hospital in your hands and those of your staff."

### A POOR-LAW PATIENT'S DONATION.

As a general rule the patient in a Poor-Law infirmary is ready to obtain cheap treatment, and should there be a loophole by which payment can be avoided it is often taken advantage of.

Boards of Guardians have their committees to consider these cases, but they often find obstacles placed in their way by the patients or their relatives. Occasionally they come across a surprise. Lambeth Board of Guardians received such a surprise when it was notified by the steward at the infirmary that he had received an anonymous registered letter containing Treasury notes to the value of £7 10s., together with an unsigned statement worded "Contributions towards Lambeth Infirmary funds." The sum was paid to the treasurer, and received by him as a thankoffering for treatment the patient had received in the infirmary. The will to give is not confined to patients of voluntary hospitals.

### THE ISSUE OF CERTIFICATES IN POOR-LAW INSTITUTIONS.

The Local Government Board have not been long in replying to the Lambeth Board of Guardians, who had declined to grant certificates to patients in this infirmary entitled to benefits under the National Insurance Act, as recorded in The Hospital on September 28. The Board consider it necessary that a person treated in a Poor-Law institution in all cases should be able to obtain, if desired, a certificate of the nature and duration of his illness; for hardship might be caused if, in the absence of such a certificate, he were unable to obtain benefits under the National Insurance Act, or as a member of a Friendly or other society. With regard to the suggestion made by the Guardians in previous correspondence, the Board pointed out that there can be no breach of medical etiquette in the giving of such a certificate to a patient, the use made of the certificate depending entirely upon the recipient. So far as cases arising under the National Insurance Act were concerned, provision had been made for dealing with cases in which it would be undesirable in the patient's interest to disclose to him his disease, and the medical superintendent at the Lambeth Infirmary, Dr. Lionel Baly, had been informed of this provision.

### THE LEGAL POSITION: BACK TO '48.

The Lambeth Board of Guardians had maintained that the National Insurance Act enforced no legal obligation on them to grant these certificates, but the Local Government Board draw their attention to the terms of Article 205 (No. 3) of the Consolidated Order of December 8, 1847, to certain parishes (including the parish of Lambeth) under separate Boards of Guardians, which regulation was still operative as regards their infirmary. This Article deals with the duties of the medical officer of a Poor-Law institution, and *inter alia* stipulates that he shall issue such certificates as the Local Government Board may from time to time decide "for the provision of medical aid and accommodation." A recent answer in the *Justice of the Peace* to a question, stated definitely that medical officers of Poor-Law institutions were legally bound to issue these certificates under the National Insurance Act when requested to do so. This settles the legal aspect of the question, with the result that the Lambeth Board of Guardians decided at once to begin again to issue the certificates. During the months in which they had suspended the practice, cases of hardship arose; unfortunately, according to Mr. Bumble, these cannot now be dealt with. Heaven knows why! But no more legal quibbling must ensue in the hope of depriving the poor of their just rights under the National Insurance or any other Act.

### THE TRICKS OF ADVERTISING.

The methods of hospital advertising have undergone much change in recent years. A bald appeal has given way to a news article, a pamphlet, even a book, and a modern hospital advertises now rather by the news of its doings than by the announcement of its needs. That is the general principle, which has been applied, so far as newspapers are concerned, to the capture of the different columns of the journals. The leading article, the "news item," the page interview, the agony column, for instance, have all been employed to secure publicity for the hospitals, and the correspondence columns of the newspapers often contain appeals more or less in disguise. The latest step seems to be an attempt to capture the customary column of snippets, generally known by some such title as "Jottings," "From Far and Near," "By Telegraph and Telephone," or, more simply, "News in Brief." Now, these potted paragraphs (to suggest a new title) frequently contain personalities. Mrs. Jones has attained her hundred-and-nineteenth birthday, Mr. Smith has been elected deputy beadle for the fourth time, the last of Mr. Robinson's nine sons has met the fate of all his brothers and hurriedly expired in great agony. And so forth. How can the hospitals capture this column? The secretary of the Great Northern Central Hospital thinks that it can be done by mentioning some of the casualties which arrive on a recent day, and giving the names of the patients. The following is a sample of his method. "On the 27th inst. G. Lyons, of Lee Green, fell from an omnibus and sustained an injury to his back; Mrs. Cook, of Pimlico, fell from an omnibus and sustained injuries to collar-bone and foot." Two more cases, occurring on the next day, are described in similar fashion, and the six-line "item" ends by stating "the injured were removed to the Great Northern Central Hospital, where they received treatment." The wonder is that the scraps which fill these columns are of the smallest interest to anybody; but since the editors of the dailies like them we suppose there is no reason why the hospitals should not insinuate themselves into these paragraphs too. What is the object gained, we hope that the secretary, Mr. Panter, will tell us.

### THE RESIDENTIAL CRECHE.

At the Mothers' Hospital, Clapton, a meeting was lately held, when Dr. William Branden, R.A.M.C., of the Clifden Road Military Hospital, presided. Since the hospital was opened by Princess Louise, four years ago, 6,656 births had been registered within its wards, and 21,461 births attended in the homes of the mothers by the district nurses. During the past year 1,000 patients had been admitted to the hospital, half the number being unmarried mothers, for whom the maternity work was originally instituted by Mrs. Booth, of the Salvation Army. The unmarried mothers are not separated from their children. To maintain their relations after the patients left the hospital, residential crèches had been planned where the mother can live with her child, go out to her daily work, and return to the child, who during her absence had been in the charge of an officer. One of these crèches is about to be opened at Highbury. There are seventy patients, and more than that number of babies, in the hospital at present. Since the war 400 soldiers' wives have been admitted. Dr. Branden stated that the Salvation Army had facilities for undertaking the care of the mother six weeks or two months before the critical event, and also for the efficient training of pupils. The maternity work began in the now demolished Ivy House. As the work developed it was found difficult to deny the advantages to married women, and the numbers of married patients were increasing so fast that it would soon be necessary to provide additional wards. The present bungalows were so constructed, according to the advice of Dr. Mackintosh, of Glasgow, as to allow another floor to be added to each without very much disturbance.

### A LITTLE JAM AT TEA-TIME.

In consequence of complaints that insufficient food was given to the wounded at the Lake Hospital, Ashton, the Board of Guardians has held an inquiry and come to the conclusion that there was nothing much the matter, though, since the hospital is rationed by the military, the committee has not so free a hand as those in charge of Red Cross hospitals. Breakfast and tea were chiefly complained of, and Mr. E. Roebuck considered that a little jam at tea-time would have sweetened the meals and the tempers of everyone. This is a point for housekeepers, whose difficulties do not end with their shopping.

### A LESSON FROM CANADA.

The Civil Re-establishment Department, 22 Victoria Street, Ottawa, is supplying information concerning the training provided by the Dominion Government for sailors and soldiers unable to resume their pre-war occupations through disabilities incurred in consequence of the war. Details concerning medical treatment supplied for illness due to military service and about the conditions under which artificial limbs and appliances are supplied and repaired are also obtainable. The question of employment is in charge of a separate decentralised department, which supplies useful information through the secretaries of the Returned Soldier Commissions of the various Provinces. We fear that the Old Country is not equally effective in the distribution of this essential detail, and as an instance of it we may remark that the official poster in respect of the efforts of Canadian authorities in this connection has reached us in London, and that its clearness and arrangement put to shame the few scattered papers which have reached us from those in charge of similar work in this country. The Ministry of Information exists; what has it done for the invalided sailors and soldiers? If their crying need to know what assistance awaits them is in charge of other central or local authorities, we should be glad to hear of them. But, in spite of a good deal that has been done by local committees, we confess a doubt as to the extent to which the need is being met.

### THE TREATMENT OF ADVANCED CASES OF TUBERCULOSIS.

Efforts are being made to induce the Local Government Board and the Metropolitan Asylums Board to place an institution at the disposal of local authorities in London for the accommodation of advanced cases of tuberculosis. In January last, at a conference of representatives of Metropolitan boroughs at the Guildhall, a resolution declared that great attention should be paid to advanced and incurable cases for which no adequate provision had been made. It was then suggested that the segregation and housing of advanced cases should be undertaken by each City and Borough Council, who could provide, in its own district, or close to its borders, a suitable home free from pauper taint. Because of the conditions due to the war this scheme is considered impracticable, but the difficulty could be surmounted if the Metropolitan Asylums Board were to devote one of their existing institutions to the isolation of these advanced cases, whether the persons were insured or not. This would reduce the cost considerably, and would obviate the difficulties that would arise if the City of London and the other twenty-eight Metropolitan boroughs were each called upon to provide accommodation for these patients.

### MATERNITY AND CHILD WELFARE COMMITTEES.

Representations have been made to the Local Government Board to the effect that pharmacists should be appointed on the committees to be established under Section 2 of the Maternity and Child Welfare Act, 1918. It is felt that the technical knowledge possessed by pharmacists would be useful to such committees in making the arrangements necessary for carrying out the intentions of the framers of the Act. The suggestion appears to be a good one, and the Board has been asked to intimate to local authorities, when sanctioning their schemes, that it is advisable that representation on the committees should be accorded to pharmacists.

### COMMISSIONED RANK AND ARMY PHARMACISTS.

Following on the decision of the Army Council to utilise enlisted pharmacists for their own skilled work in the Army, the War Office has now informed the Pharmaceutical Society of Great Britain that it proposes to issue an "Army Council Instruction" to the effect that, in future, a "duly qualified pharmacist" shall be in charge of the dispensary of every military hospital with 100 beds or more. Pharmacists acting in this capacity are to rank as sergeants. The question of commissioned rank, and also higher rank than sergeant, is still under consideration by the Director-General, Army Medical Service, who will issue his decision on this matter later. It is also satisfactory to note that *all* pharmacists who now enlist will be posted to the R.A.M.C. for dispensing or chemical work as required. The Pharmaceutical Council is to be congratulated on the result of its persistent efforts to secure an adequate status to its members engaged on military work, whilst the sick and wounded should benefit by a more efficient dispensing service.

### THE NEW SALARIES FOR DISPENSERS.

The Council of the Public Pharmacists' Association recently decided not to arrange any lectures or social functions for the coming winter, because of the fact that most of the members are engaged in "war" work of various kinds. The Council will, however, meet, as occasion demands, to deal with matters affecting the welfare of pharmacists engaged in hospital and institutional dispensing. Amongst other matters dealt with by the Council was a report on the result of an appeal to the Local Government Board to have the scale of salary for dispensers increased, and, whilst no concession had been made as regards raising the initial, *commencing* salary, the Local Government Board had agreed to increases at more frequent intervals and to make the maximum salary £200 per annum without stipulating, as previously, that any special circumstances must exist to ensure this amount. Many women pharmacists engaged in hospital work have joined the Association, and they have a representative on the Council, Miss Andrews, to help in dealing with any matters specially affecting women dispensers. A special resolution of regret at the death of Mr. W. E. Miller was recorded on the minutes and a vote of condolence passed to Mrs. Miller and to his family. Action taken regarding dispensing work in the Navy and Army was reported and approved. The result of negotiations with the Army Council was received with satisfaction as an earnest of more reforms to follow in the near future. The chairman, Mr. A. H. Jenkin, expressed the view that many changes were in prospect which would affect public pharmacists in common with other professional workers, and that all pharmacists engaged in institutional work should join an association to protect their professional interests.

### MUNICIPAL SALARIES AND DUTIES.

At the Willesden Municipal Hospital, Mr. Norman Patterson, F.R.C.S., has been appointed as throat, nose and ear specialist on terms arranged at a salary of 125 guineas per annum. Dr. Mabel Campbell Clarke, the junior resident medical officer, has resigned, and Miss Isabella McFadyean has been appointed. Dr. Estelle Atkinson, a permanent assistant medical officer, who has £438 a year, has asked for the same payment as the temporary officer, namely, £547 10s. Having regard to her qualifications, experience, and length of service, the Health Committee suggests that her salary be increased to £600, and that a deduction of £150 a year be made while she is in receipt of board, lodging and clothing at the hospital. The medical officer has been instructed to apply to the Education Committee on behalf of the health visitors at the municipal clinic for temporary arrangements to be made for their meals to be supplied at the Strode Road feeding centre. The medical officer responds that provision has been made for nursing and medical attendance in 212 cases of confinement at home and fifty-one at hospital since the opening of the municipal clinics and maternity pavilion respectively. The Health Committee has had an application from the twenty-six health visitors for an increase of £20 a year, and recommends acquiescence with the request.

### "BLOSSOM BY BLOSSOM THE SPRING BEGINS."

In our recent review of Mr. Laurence Binyon's description of British Red Cross activities in France, we alluded to the reconstruction of the ruined villages, which too often have been the beach on which the tide of devastation has ebbed and flowed. In the past, these villages after their rescue, till the superb advance of the past two months, have passed again under the German heel. The fate of such villages is hardly to be thought on. It is therefore good to read the following terse statement in a very recent issue of the *American Red Cross Bulletin*. "Arrangements are being made by the American Red Cross to send out detachments of workmen for the provisional repair of the shell-ruined villages reclaimed by the recent attacks in the American zone in France." As the spring returns undismayed, even in the muddy districts of Flanders, so does the permanent spring of human and constructive effort survive every attempt to overwhelm the upthrust of its life. "A little while and ye shall not see Me, and again a little while and ye shall see Me." It is the word "again" which the Allied victories are now causing in these thrice-ruined villages to be heard.

### THIS WEEK'S DRUG MARKET.

How are the developments in the direction of peace affecting the markets? This is a question that is being asked not only in the drug markets, but in practically all markets. So far as drugs are concerned the immediate results are not very apparent, although there is a distinct tendency among buyers to proceed cautiously and not to do to-day what can be done to-morrow. The substantial advance in the price of eucalyptus oil is not due to the war situation, but to the fact that the demand is above the normal and the shipments from Australia much below the normal. Star aniseed oil is dearer. The price of aspirin is maintained. Bromides are steady, but it is hoped that supplies will be coming from America more freely. Japanese crude camphor is much dearer, and English refiners have withdrawn their quotations. Chloral hydrate tends downwards in price. Citrates are much dearer. A good business has been done in lemon oil at firmly maintained rates. Menthol is again rather dearer. Saccharin has a lower price tendency, and the prices of salicylates are not quite so firmly maintained.

